# Impact of a High Protein Intake on the Plasma Metabolome in Elderly Males: 10 Week Randomized Dietary Intervention

**DOI:** 10.3389/fnut.2019.00180

**Published:** 2019-12-06

**Authors:** Brenan Durainayagam, Cameron J. Mitchell, Amber M. Milan, Nina Zeng, Pankaja Sharma, Sarah M. Mitchell, Farha Ramzan, Scott O. Knowles, Anders Sjödin, Karl-Heinz Wagner, Nicole C. Roy, Karl Fraser, David Cameron-Smith

**Affiliations:** ^1^Liggins Institute, University of Auckland, Auckland, New Zealand; ^2^Division of Systems Medicine and Digestive Medicine, Department of Metabolism, Digestion and Reproduction, Imperial College London, London, United Kingdom; ^3^School of Kinesiology, The University of British Columbia, Vancouver, BC, Canada; ^4^Food Nutrition & Health Team, AgResearch, Palmerston North, New Zealand; ^5^Department of Nutrition, Exercise and Sport, Copenhagen University, Copenhagen, Denmark; ^6^Department of Nutritional Sciences and Research Platform Active Ageing, University of Vienna, Vienna, Austria; ^7^The High-Value Nutrition National Science Challenge, Auckland, New Zealand; ^8^Food & Bio-based Products Group, AgResearch, Palmerston North, New Zealand; ^9^Riddet Institute, Massey University, Palmerston North, New Zealand; ^10^Clinical Nutrition Research Centre, Singapore Institute for Clinical Sciences, Agency for Science, Technology, and Research, Singapore, Singapore

**Keywords:** dietary protein, plasma metabolomics, older adults, pathway mapping, nutritional interventions

## Abstract

High protein diets may improve the maintenance of skeletal muscle mass in the elderly, although it remains less clear what broader impact such diets have on whole body metabolic regulation in the elderly. Non-targeted polar metabolomics analysis using HILIC HPLC-MS was used to profile the circulating plasma metabolome of elderly men (*n* = 31; 74.7 ± 4.0 years) who were randomized to consume for 10 weeks a diet designed to achieve either protein (RDA; 0.8·g^−1^·kg^−1^) or that doubled this recommend intake (2RDA; 1.6.g.kg^−1^). A limited number of plasma metabolites (*n* = 24) were significantly differentially regulated by the diet. These included markers of protein anabolism, which increased by the 2RDA diet, including; urea, creatine, and glutarylcarnitine. Whilst in response to the RDA diet; glutamine, glutamic acid, and proline were increased, relative to the 2RDA diet (*p* < 0.05). Metaboanalyst identified six major metabolic pathways to be influenced by the quantity of protein intake, most notably the arginine and proline pathways. Doubling of the recommended protein intake in older males over 10 weeks exerted only a limited impact on circulating metabolites, as determined by LC-MS. This metabolomic response was almost entirely due to increased circulating abundances of metabolites potentially indicative of altered protein anabolism, without evidence of impact on pathways for metabolic health.

**Trial Registration:** This trial was registered on 3rd March 2016 at the Australia New Zealand Clinical Trial Registry (www.anzctr.org.au) at ACTRN 12616000310460.

## Background

An adequate intake of dietary protein is essential for the maintenance of health in the elderly. Yet considerable debate exists as to how much protein is required for maintaining optimal health in the aging population (1). Recent analysis has shown that dietary intakes only meeting the World Health Organization (WHO) recommended daily allowance (RDA) for dietary protein (0.8 g·kg^−1^·d^−1^) increase the risk of progressive skeletal muscle and muscular strength and/or function loss in the elderly (2–4). This loss of muscle mass and function, known as sarcopenia, is a major risk factor for the loss of independence, increasing the likelihood for institutionalized care and mortality risk (5). Recent meta-analysis of both intervention and cohort studies provides additional evidence that diets that have protein intakes greater than the RDA offer protection against muscle loss in the elderly (6, 7). Recent expert opinions and consensus statements have therefore suggested that older people should be aiming to consume diets that provide well in excess of the RDA, with recommendations ranging from 1.0 to 1.5 g·kg^−1^·d^−1^ (8– 10).

Whilst there is emerging evidence of the potential benefits of higher intakes of protein for maintenance of muscle mass and function, the value and importance of dietary protein in the context of various changes in digestive and metabolic function, and the associated changes in liver and renal function, in the elderly, require careful consideration (11, 12). It must be considered whether elevated protein impacts on the already established heightened risk and a wide range of complex diseases, including many cancers, type 2 diabetes and cardiovascular disease, that is experienced in the elderly (13, 14). To date, analysis of the impact of dietary protein in the elderly on metabolic disease risk is limited, with studies demonstrating an adverse effect of diets rich in animal proteins, with the possible exception of dairy protein (15, 16).

In order to define evidence-based protein intake recommendations, it is therefore important to comprehensively understand the metabolic implications of an increase in dietary protein intake. These metabolic adaptations are undoubtedly complex, as altered protein intake impacts on digestive hormones, circulating amino acids and their metabolites, but also exerts an as yet poorly defined impact on the human gut microbiome (16, 17). A range of nutritional intervention studies have utilized either targeted or untargeted metabolomics approaches to profile the complex metabolic adaptations to diet (18–20). Profiling of either plasma or urinary metabolites has been able to identify major features of metabolic adaptation and possible mechanisms by which health status is altered (12, 21). These studies have also provided significant insight into a range of metabolites that form predictive biomarkers of dietary intake and habits that can be subsequently applied to the analysis of habitual diet or adherence to a dietary intervention (22, 23).

Recently, we have reported on the primary outcomes of a 10-week intervention altering protein intake in older men, in which it was demonstrated that a controlled whole food diet intervention that doubled the WHO recommendation resulted in reduced loss of skeletal muscle size and function, than a diet only meeting the WHO protein recommendation (7). Thus, in a secondary analysis, this study aimed to further examine the impacts of a high protein diet on metabolic function using a global/untargeted metabolomics analysis of polar metabolites via hydrophilic interaction liquid chromatography-mass spectrometry (HILIC-MS) (24). It is hypothesized that a 10 week diet that doubles the RDA for protein (2RDA: 1.6 g·kg^−1^·d^−1^) in comparison to a diet that meets the RDA (RDA: 0.8 g·kg^−1^·d^−1^) would result in a significant alteration of polar metabolites related primarily to amino acid oxidation and anabolism, with additional adaptation in pathways related to metabolic regulation of carbohydrate and lipid metabolic homeostasis.

## Methods

### Ethics Statement

Written informed consent was obtained from all participants before participation in the trial. The study was reviewed and approved by the Southern Health and Disability Ethics Committee (New Zealand; 15/STH/236) and was conducted in accordance with the Declaration of Helsinki. The study was prospectively registered with the Australian and New Zealand Clinical Trial Registry (www.anzctr.org.au) as ACTRN12616000310460 on March 9, 2016.

### Participants

Healthy elderly men, ≥70 years were recruited through advertisements to participate in the study. All participants were free-living individuals who had a body mass index (BMI; kg/m^2^) ranging from 18 to 35. All were non-smokers who did not consume any dietary supplements 1 month before participating in the study. Exclusion criteria to those potential participants were those who consumed a restricted diet, including vegetarians or with self-reported food allergies (e.g., nuts, fish, dairy). Further exclusion was applied to those with a prior history of cancer, diabetes, thyroid disease or conditions affecting neuromuscular function.

### Experimental Design

The design of this study has been previously described in Mitchell et al. (7). Participants were randomized into two groups (equal allocation ratio) where they either received a controlled diet of 0.8 protein kg^−1^·d^−1^ (RDA) or 1.6 g protein kg^−1^·d^−1^ (2RDA) for 10 weeks. All meals consumed by the participants during the 10-week study were provided. Energy from fat was standardized (28–31% of energy), with the difference being made up of carbohydrates. All diets adhered to Eating and Activity Guidelines for New Zealand (25). Adults met the recommendations for intake of fruit and vegetables. All participants completed dietary records to ensure all food provided was consumed, and food selection was adjusted to participants' preferences to maintain high compliance. Any non-study food consumed was also asked to be recorded. The energy content of the intervention diet was individually calculated to match participants estimated energy needs based on the Harris-Benedict equation (26) and adjusted for physical activity, which was assessed by wrist-worn accelerometers (Fitbit Charge HR). The estimated energy needs were calculated before the intervention and adjusted fortnightly based on participant weight maintenance and satiety to ensure participants consumed adequate protein relative to energy intake. Throughout the study protein and energy were distributed between breakfast, lunch and dinner as 30, 30, and 40%, respectively. During the intervention, participants were instructed to maintain their normal lifestyle, and prepared meals were delivered to their homes. All testing was conducted at the University of Auckland Nutrition and Mobility Clinic.

### Metabolomics Analysis

Fasted plasma was collected pre-intervention and at the completion of 10 weeks of diet intervention. Extraction of metabolites was performed using a previously described bi-phasic method (27). Briefly, 100 μl of plasma was mixed with 800 μl of cold (−20°C) CHCl_3_/MeOH (50:50, v/v), agitated for 30 s and stored at −20°C for 60 min to allow for protein precipitation. Subsequently, 400 μl water was added, the mixture was vortexed (2 × 15 s) and centrifuged for 10 min at 14,000 rpm and 4°C to separate the aqueous (upper) and organic (lower) phases.

The lower organic phase is the lipid portion of the extract and was not analyzed as the interest in this clinical trial was around the impact of prolonged increased protein intake on the metabolome. Thus, any changes are likely to occur in the polar metabolome rather than the lipidome.

The aqueous phase (200 μl) was collected, dried under a stream of nitrogen and reconstituted in 200 μl of acetonitrile: water (50:50, v/v) containing 10 μg/ml d_2_-tyrosine as an internal standard. Samples were placed in the LC-auto-sampler at 4°C for HILIC-MS analysis. Blank procedure samples were prepared exactly as the samples, but plasma was replaced with Milli-Q water. To avoid any systematic analytical effects, samples were randomized before analysis. To verify and/or maintain data quality within each mode, a QC sample (comprising a pooled extract of all samples) was also injected once for every 10 samples. Retention time, signal/intensity, and mass error of internal standards were monitored constantly to check instrument response variability and retention time shifts.

Plasma extracts were analyzed by HILIC LC-MS (Thermo Fisher Scientific, Waltham, MA, USA) using both positive and negative ionization modes. HILIC-MS conditions have been previously described in Fraser et al. (24). Briefly, compounds were separated using a 5 μm ZIC-pHILIC column (100 × 2.1 mm, 5 μm; Merck Darmstadt, Germany) eluted with solvent A: acetonitrile with 0.1% formic acid, and solvent B: 16 mM ammonium formate in water, at a flow rate of 250 μl/min. Initial conditions of the solvent gradient were set at 97:3 (A:B), which was held for 1 min, where after that the gradient was changed linearly to give a ratio 10:90 (A:B) at 14.5 min and maintained till 17 min. At this time and up to 24 min, the column was allowed to return to initial conditions and re-equilibrate. The column effluent was connected to an electrospray source of a high-resolution mass spectrometer (Exactive Orbitrap, Thermo, San Jose, CA, USA). Mass spectral data were collected in profile data acquisition mode covering a mass range of *m/z* = 55–1,100 with a mass resolution setting of 25,000 and a maximum trap fill time of 250 ms using the Xcalibur software package (Thermo, San Jose, CA, USA).

Data processing consisted of a series of procedures aimed at converting raw mass spectrometry data to data matrices suitable for further statistical analyses. Peak detection, alignment, grouping, and noise elimination were performed using XCMS software (28). The resultant peak intensity table was subjected to run-order correction and batch normalization utilizing pooled QC samples and applying the lowess regression model (29). This was evaluated on the open-source online platform Workflow4metabolomics (https://workflow4metabolomics.org) (30). Finally, features with a CV of >30% within the pooled QC samples were removed.

### Statistical Analysis

Principal component analysis (PCA) was used to validate the quality of the analytical system performance and observe possible outliers using the commercial package SIMCA-P+ version 14.1 software (Umetrics, Umea, Sweden). Two-way repeated measures ANOVA was used to determine differences in groups with time (pre vs. post) as a repeated factor and diet as a fixed factor using R (version 3.4.2). *Post-hoc* comparisons were conducted by Holm-Sidak corrections. False discovery rate adjustment of *P*-values (FDR) was applied using the Benjamini-Hochberg method.

### Peak Identification

Peak identification was performed by verifying the accuracy of mass measurements, the retention time and the tandem mass spectrometry results against an in-house database, which contained over 600 authentic standard compounds. Where a feature did not match the library, the molecular and co-eluting ions were manually inspected. The source voltage applied created a small amount of source induced fragmentation which could be used to facilitate annotation of the unknown features. Thus, pseudo MS^2^ spectral data was validated by inspecting each metabolite for perfectly co-eluting fragment and molecular ions. These were matched to the MS/MS data provided on public libraries such as METLIN (31) and the Human Metabolome Database (HMDB) (32). The use of pseudo MS^2^ spectral data to validate the molecular ion has been previously described as an acceptable approach (33) and has been applied in large cohort trials such as the HUSWERMET project (34). Each metabolite was assigned a confidence level defined by the Metabolomics Standard Initiative (35). Therefore, the level 2 annotation is based on co-eluting accurate mass fragment ions observed with the accurate mass of the molecular ion.

### Network Analysis and Metabolic Pathway Construction

MetaboAnalyst (version 4.0) was used for pathway enrichment analysis utilizing the Kyoto Encyclopedia of Genes and Genomes (KEGG) database, which can provide valuable information by integrating two pathway analysis approaches; pathway enrichment analysis and pathway topology analysis (32). The hypergeometric test was selected to evaluate whether a particular metabolite set was represented more than once (36). The relative-betweenness centrality algorithm was applied for pathway topology analysis measuring the numbers of shortest paths going through a node in the network. This takes into consideration the global network structure, not only the immediate neighbor of the current node (37). The acquired impact value represents the accumulative percentage of importance for the matched metabolite nodes involved in a pathway. Network construction was performed by Cytoscape (version 3.4.0) (38) utilizing the plugin Metscape and topological parameters were analyzed using the network analyser tool (39). To confirm the most important metabolites of the network, two well-established node centrality measures were used to estimate node importance; the degree of each node and betweenness.

## Results

### Subject Characteristics and Dietary Intake

The participants were randomly assigned to either the RDA (*n* = 16) or 2RDA (*n* = 15) diet; of these one subject withdrew his consent before the start of the dietary intervention (RDA group) and one subject from the 2RDA group did not complete the 10-week intervention due to failed diet compliance (Table 1). Based on dietary records, compliance was 98.9% for both protein and energy intake in the RDA group and 97.5% and 98.4% for protein and energy intake, respectively in the 2RDA group, with no compliance difference between the two groups. As previously reported, both groups shared a small energy deficit of 209 ± 213 and 145 ± 214 kcal/d in the RDA and 2RDA groups, respectively, with no energy deficit difference between the two groups. Detailed changes in body composition and macronutrient information can be found in Mitchell et al. (7).

**Table 1:** Physical characteristics of participants.

	**RDA (*n* = 15)**	**2RDA (*n* = 14)**
Age (years)	74.9 ± 1.1	73.8 ± 0.9
Height (cm)	171.8 ± 2.0	172.1 ± 1.6
Weight (kg)	83.9 ± 5.4	83.0 ± 2.4
BMI (kg/m^2^)	28.2 ± 1.4	28.0 ± 0.9

*Values are means ± standard error of the mean. The RDA is 0.8 g protein·kg^−1^·d^−1^, 2RDA is 1.6 protein·kg^−1^·d^−1^. BMI, Body mass index, RDA, Recommended dietary allowance*.

### Metabolomics Analysis

**Figure 1:**
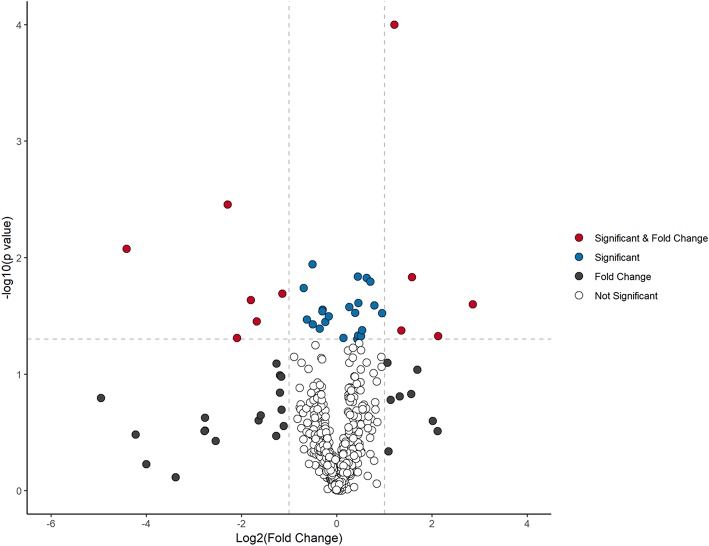
Volcano plot analysis of metabolite changes after 10 weeks dietary intervention. Red dots denote significant (*p* < 0.05) and fold change (>1.2) features. Blue dots represent significant features (*p* < 0.05), black dots designate fold change (>1.2), and clear dots represent neither no significance (*p* > 0.05) or fold change of <1.2.

The stability and reproducibility of the current data were evaluated by the QC samples measured during the whole experimental period. PCA score plot representation analysis on the whole data matrix, which included all features (*m*/*z* and retention time) showed no obvious run-order effects for metabolite profiles obtained in positive and negative ion modes (Supplementary Figure 1). This result demonstrated good stability and reproducibility in the current metabolomic dataset. After data processing, 170 and 275 features were detected in positive and negative ionization mode respectively, which were pooled together to visualize in a volcano plot (Figure 1). The total number of features (445) were separated by significance (post-intervention *t*-test) and fold change (>1.2).

**Figure 2:**
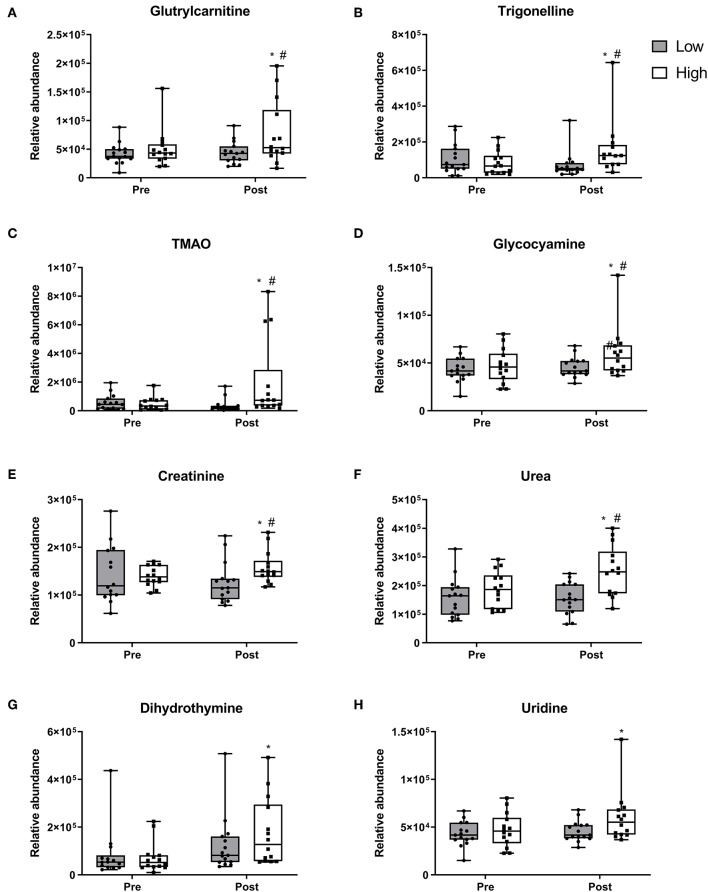
Metabolites which increased in the 2RDA diet. Box and whisker plot showing median, first, and third quartiles, and maximum and minimum values. **(A)** glutrylcarnitine, **(B)** trigonelline, **(C)** TMAO, **(D)** glycocyamine, **(E)** creatinine, **(F)** urea, **(G)** dihydrothymine, and **(H)** uridine increased within the 2RDA group. ^#^denotes the significant differences of metabolites between 2RDA and RDA. *Represents the significant differences of metabolites between pre and post intervention within each group.

Two-way repeated measure ANOVA revealed 24 metabolites exhibiting changes between and within diet groups (*p* < 0.05). These differentially regulated metabolites are classified by using the four levels of certainty as defined by Sumner et al. (35) (Supplementary Table 1). After 10 weeks, nine metabolites demonstrated increases only within the 2RDA group. Glutrylcarnitine (Figure 2A), trigonelline (Figure 2B), trimethylamine *N*-Oxide (TMAO; Figure 2C), glycocyamine (Figure 2D), creatinine (Figure 2E), and urea (Figure 2F) all increased within the 2RDA group (*p* < 0.05 each, respectively), whereas there were no changes in the RDA group for these metabolites. Furtheremore, these metabolites were significantly elevated in the 2RDA group compared to the RDA group post-intervention (*p* < 0.05 each, respectively). Two pyrimidines, dihydrothymine (Figure 2G) and uridine (Figure 2H), along with an unknown metabolite (Supplementary Figure 2A) increased within the 2RDA group after 10 weeks but were not higher than the RDA group at the post-intervention stage (*p* < 0.05 each, respectively).

**Figure 3:**
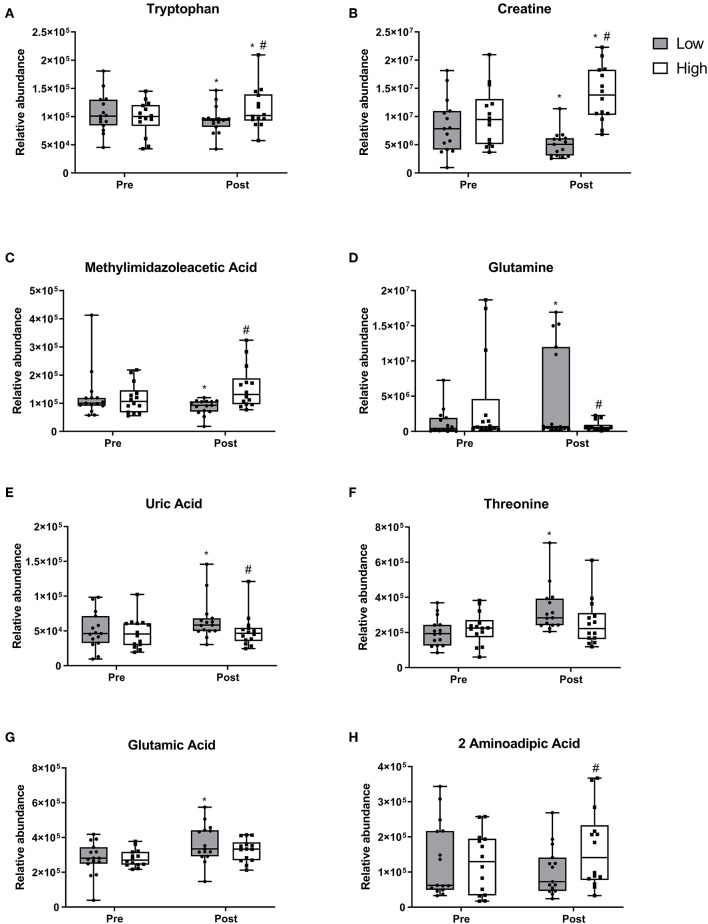
Metabolite responses to 2RDA or RDA after 10 week intervention. Box and whisker plots showing median, first, and third quartiles, and maximum and minimum values. **(A)** tryptophan, **(B)** creatine, **(C)** methylimidazoleacetic acid increased in the 2RDA group. **(D)** glutamine **(E)** uric acid, **(F)** threonine, **(G)** glutamic acid all increased in the RDA group. **(H)** 2-aminoadipic acid was higher in the 2RDA group compared to the RDA group. ^#^denotes the significant differences of metabolites between 2RDA and RDA. *Represents the significant differences of metabolites between pre and post intervention within each group.

Ten metabolites demonstrated changes within the RDA group after the 10 week intervention. Tryptophan (Figure 3A), creatine (Figure 3B) and methylimidazoleacetic acid (Figure 3C) all reduced within the RDA group and post-intervention were lower in the RDA group compared to the 2RDA group (*p* < 0.05 each, respectively). Furthermore, the 2RDA diet increased the level of tryptophan (*p* = 0.04) and creatine (*p* = 0.001) after the 10 week intervention. Glutamine (Figure 3D), uric acid (Figure 3E) and unknown metabolite M160T639 (Supplementary Figure 2B) increased within the RDA group with not changes obeserved within the 2RDA group (*p* < 0.05 each, respectively). Post-intervention, the RDA group was higher than the 2RDA group for uric acid (*p* = 0.02) and glutamine (*p* = 0.03). Threonine (Figure 3F) and glutamic acid (Figure 3G) along with unknown metabolite M145T531 (Supplementary Figure 2C) increased only within the RDA group with no difference observed between groups post-intervention (*p* < 0.05 each, respectively). 2-aminoadipic acid (Figure 3H) did not show any changes within diet groups but was elevated in the 2RDA group compared RDA group post-intervention (*p* = 0.04).

**Figure 4:**
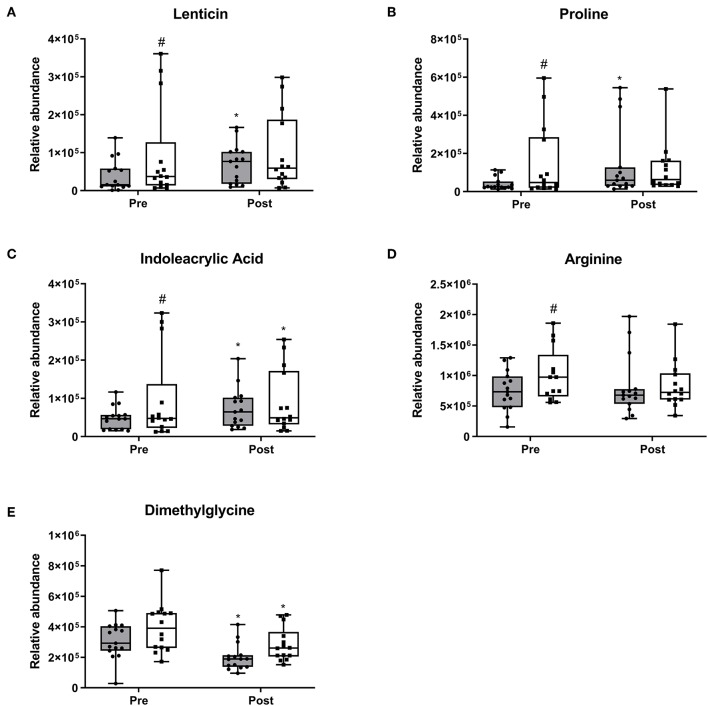
Pre-intervention metabolite differences. Box and whisker plots show median, first, and third quartiles, and maximum and minimum values. There were four metabolites which exhibited pre-intervention differences **(A)** lenticin, **(B)** proline, **(C)** indoleacrylic acid, and **(D)** arginine. **(E)** dimethylglycine decreased in both diet groups. ^#^denotes the significant differences of metabolites between 2RDA and RDA. *Represents the significant differences between pre and post-intervention within each group.

At baseline, four metabolites were different among the 30 participants, which converged at the end of the 10-week period. Lenticin (Figure 4A) and proline (Figure 4B) increased within the RDA group (*p* < 0.05 each, respectively). Indoleacrylic acid increased within both the RDA (Figure 4C; *p* = 0.03) and 2RDA group (*p* = 0.003). Arginine (Figure 4D) was also different at baseline (*p* = 0.02) but demonstrated no changes within diet and at the post-intervention stage. Dimethylglycine (Figure 4E) decreased in both diet groups with no pre or post difference (*p* < 0.05 each, respectively).

### Pathway Analysis

Six pathways were statistically significant as determined by MetaboAnalyst; five pathways relating to amino acid metabolism are ranked in descending order in Table 2. Arginine and proline metabolism was the most significant with eight identified metabolites. The pathways were composed of metabolites that increased within the RDA group such as; glutamine, glutamate, and proline. In addition, metabolites that increased within 2RDA group; urea, glycocyamine, creatinine, creatine, and tryptohan were also important components in the pathways identified.

**Table 2:** Description of the total number of compounds in the pathway.

**Pathway name**	**Total compound^a^**	**Hits^b^**	**Raw p^c^**	**–Log (p)**	**Impact^d^**
Arginine & proline metabolism	77	8	2.57E-08	2.06E-06	0.356
Aminoacyl-tRNA biosynthesis	75	6	1.03E-05	0.00041	0.0563
Glycine, serine & threonine metabolism	48	5	1.80E-05	0.00048	0.137
Pyridine metabolism	60	4	0.00821	0.0164	0.0355
Nitrogen metabolism	39	3	0.00271	0.0372	0.000
Glutamine & glutamate metabolism	11	2	0.00279	0.0372	0.139
Alanine, aspartate & glutamate metabolism	24	2	0.0132	0.151	0.384

a *Total compound is the number of compounds involved in the pathway*.

b *Hits is the matched number from the user uploaded data*.

c *The raw p is the original p-value calculated from the enrichment analysis*.

d *Impact value is calculated from pathway topology analysis for comparison among different pathways. It represents the cumulative percentage of importance for the matched metabolite nodes involved in a pathway. The importance of each metabolite node is calculated from centrality measures and represents the percentage with regard to the total pathway importance*.

### Metabolic Network Analysis

**Figure 5:**
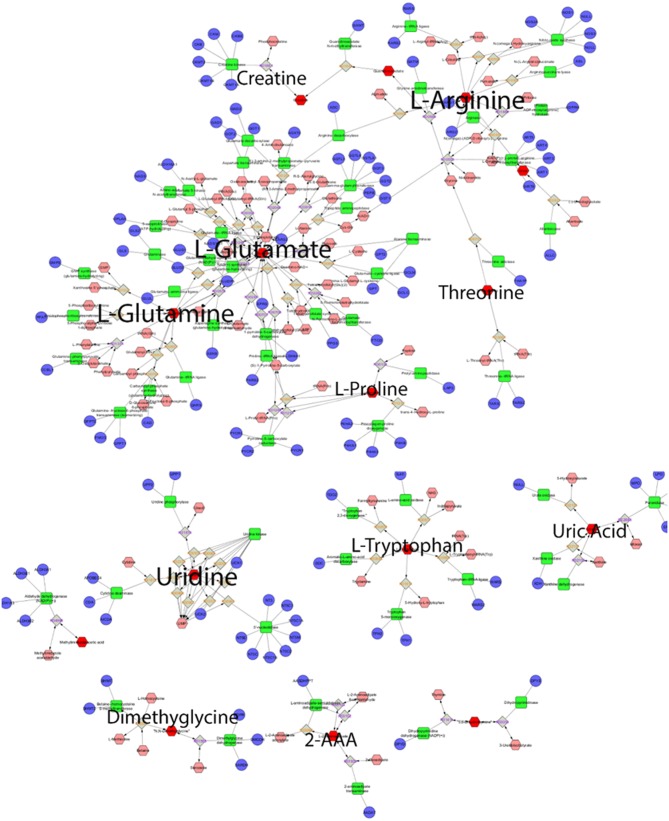
Global metabolic network pathways. Nodes correspond to the identified plasma metabolite, and edges indicate a significant correlation between nodes. Red hexagons represent inputted metabolites; pink hexagons represent compounds, blue circles represent genes, green quadrangles represent enzymes, gray quadrangles represent reaction paths.

Figure 5 displays a network visualization, where it demonstrates the centrality of glutamine, glutamic acid, arginine, and uridine and their connectivity to an array of metabolites and enzymes. These metabolites are considered hub metabolites based on measures of degree >10 and betweenness centrality (>0.1), where they are not only components of pathways but are also integral to pathway performance (Table 3).

**Table 3:** Topological parameters of key metabolites.

**Metabolite**	**KeggID**	**Degree**	**Betweenness centrality**	**Pathways**
Glutamic acid	C00025	39	0.670	Arginine & proline metabolism, Aminoacyl-tRNA biosynthesis, Nitrogen metabolism, Glutamine & glutamate metabolism, Alanine, aspartate & glutamate metabolism
Glutamine	C00064	19	0.645	Arginine & proline metabolism, Aminoacyl-tRNA biosynthesis, Nitrogen metabolism, Glutamine & glutamate metabolism, Alanine, aspartate & glutamate metabolism, Pyrimidine metabolism
Arginine	C00062	16	0.444	Argine & proline metabolism, Aminoacyl-tRNA biosynthesis
Uridine	C00299	12	1.000	Pyrimidine metabolism

*The degree of a node is the number of edges associated with it, and the betweenness centrality of a node is the number of shortest communication paths between different pairs of nodes*.

## Discussion

Comprehensive polar metabolomics analysis via HILIC-LCMS was applied to the plasma samples from elderly males who consumed a diet that contained foods to achieve the RDA for protein or when the diet was manipulated to achieve twice the RDA (2RDA). The key finding was centered on amino acid metabolism, with very few significant changes in metabolites not directly related to amino acid metabolism. Eleven metabolites were different between the RDA and 2RDA groups at the end of the interevention. Adherence to a diet that met the minimum dietary recommendations (RDA) resulted in increased circulatory concentrations of a limited subset of non-essential amino acids, including; glutamine, glutamic acid, and proline. These amino acids, along with arginine, have been identified as integral hubs where metabolic pathways overlap and may be indicative of increased overall tissue catabolism. With an increased protein consumption (2RDA), there were alterations in the circulatory metabolome consistent with increased amino acid flux and nitrogenous oxidative metabolism. There was also a shift in circulating metabolites that may be indicative of induction of anabolic pathways (40, 41). This metabolomic analysis provides evidence of how changes in dietary protein may impact the regulation of muscle mass. In this study, these results provide support for our previously published findings of increased muscle mass and physical strength in the older men who consumed the higher protein diet (7).

### Arginine and Proline Metabolism

Greatest statistical discrimination between the diets related to arginine and proline pathways, which comprised of eight metabolites with fold changes ranging between 1.2 and 2.7. The high centrality of arginine places it as a key metabolic hub, acting as a precursor for downstream targets. In this pathway, arginine is converted by arginase-1 to urea and is also the precursor to creatine through the intermediate glycocyamine. Urea and creatine are widely used markers of protein intake (42). Arginine is also important in overall skeletal muscle protein balance as a key substrate for nitric oxide, an endogenous regulator of vascular homeostasis (43). Evidence suggests nitric oxide is integral to skeletal muscle contractile function, blood flow, and glucose homeostasis, further highlighting the importance of arginine (44). Also part of the arginine-proline pathway is glutamic acid, the precursor to proline, which increased 3-fold in the RDA group. High levels of proline have been previously implicated as a risk factor for sarcopenia and frailty (45). Therefore, in the current study, the post-intervention difference between diet groups by the observed approximate 30% elevations in urea, glycocyamine, and creatine in the 2RDA group together with the alterations in the arginine and proline pathways, is indicative of a metabolomic milleau potentially favorable to sustaining anabolism.

### Glycine, Serine, and Threonine Metabolism

Five metabolites from the glycine, serine, and threonine metabolism pathway were regulated by protein intake, including; glycocyamine, creatine, and tryptophan all of which were higher in the 2RDA group compared to the RDA group. Of these, it has been shown that glycine, serine, and threonine metabolism are central to providing precursors for the synthesis of proteins, nucleic acids, and lipids (46). Threonine was increased by 60% in the RDA group and has been shown to follow the changes of branch chain amino acids (BCAAs) in situations of muscle catabolism (47, 48). Dimethylglycine which was identified to be part of the same cycle is produced from betaine and metabolized to glycine, decreased in both RDA and 2RDA groups. Dimenthylglycine is a degradation product for one-carbon metabolism, which supports multiple physiological processes such as; amino acid metabolism, and biosynthesis of purines (46). Tryptophan has multiple biological roles and whilst it has not been directly implicated in tissue anabolism, it is the precursor of serotonin and reflected in mood and cognition (49). Therefore, while its role is difficult to ascertain, tryptophan's fasting plasma levels may be of significant interest as an indicator of nutritional status beyond anabolism. Contrasting observations have been reported for relationships between tryptophan and skeletal muscle. Moaddel et al. (50) associated higher levels of tryptophan with low muscle quality, while in Toyoshima et al. (45) tryptophan was lower in sarcopenic participants. Our findings for tryptophan are in agreement with Toyoshima et al. (45) where the 2RDA group increased the plasma level of tryptophan by 16% and was higher than the RDA group at the end of the intervention.

### Aminoacyl-tRNA Biosynthesis, Nitrogen Metabolism, and Pyrimidine Metabolism

Metabolism of the aminoacyl-tRNA biosynthesis and nitrogen metabolism pathway also discriminated between 2RDA and RDA with six and three identified metabolites, respectively. Glutamine and glutamic acid are components of both aminoacyl-tRNA and nitrogen metabolism, which along with threonine, proline, and arginine (which demonstrated a trend), increased in the RDA group. Aminoacyl-tRNA substrates are the essential first steps for protein synthesis. Dysregulation of these pathways has been linked with aging muscle (51). Based on our previous demonstration of reduced appendicular lean mass in the RDA group (7), these data suggest the circulatory alterations both aminoacyl-tRNAs and nitrogen metabolism are reflective of this loss of muscle mass.

Uridine, dihydrothymine, and urea, were metabolites identified to be components of pyrimidine metabolism where glucose and glutamine serve as precursors. These metabolites were each increased in the 2RDA group, by at least 60%. Pyrimidine metabolism, is integral to cell cycle regulation, and tightly linked to the ability of the cell to acquire nutrients, generate metabolic energy, and to drive anabolism, including nucleotide/nucleic acid biosynthesis (52). Glutamine is an important component of muscle protein, and helps repair and build muscle (53). Furthermore, glutamine deficiency stimulates cell apoptosis, which may thereby trigger a low-level inflammatory process and increase the rate of sarcopenic progression (54). In times of catabolic stress, glutamine derived by *de novo* synthesis from skeletal BCAAs are used to form glutamic acid. Glutamic acid displays remarkable metabolic versatility being highly abundant in liver, kidney, skeletal muscle, and the brain, illustrating its utility (55). In this study, glutamine increased 3-fold and glutamic acid was elevated by 30% in the RDA group. which is concomitant with physical changes of reduced lean muscle (7).

### Markers of Specific Diet Consumption

The findings of the current study are complex in that there were observed baseline differences revealed by the 2-way repeated measures ANOVA, prior to the dietary interventions, in a small subset of metabolites. This was despite the cohort being relatively homogenous in terms of age, BMI, background diet and general metabolic and cardiovascular health. It is unclear whether these differences are then reflective of the complexity associated with inter-individual variability or indicative of a difference in habitual diet or associated lifestyle factor(s). Notable amongst the pre-intervention differences was lenticin, found in lentil extracts and is detected in blood after lentil consumption (56). These observed baseline differences were normalized with the 10 week intervention, as the participants adhered to the well-designed meals matched for micronutrients, macronutrients, and food structure, except protein.

In this study, uric acid, the end product of purine metabolism, was increased by 30% in the RDA diet. Increased concentrations of uric acid is a causative factor in gout (57), where previously, it has been postulated that higher consumption of dietary protein increases uric acid excretion, thereby reducing circulatory uric acid (58, 59). TMAO is generated from choline, betaine, and carnitine by gut microbial metabolism, increased in the 2RDA group by 5-fold with no change in the RDA group. The specific increase of TMAO in the 2RDA is similar to previous observations of well-known protein sources; fish (60) and meat (61). Whilst, the role of TMAO remains speculative toward gut health and CVD risk (62), the present finding suggests it can be regarded as a marker of high protein intake.

### Limitations

This trial applied an untargeted HILIC-MS approach to cover polar metabolites, yet the metabolome is vast in number and diverse in nature. There is opportunity for future studies to implement more comprehensive untargeted metabolomics analysis to cover greater diversity of the metabolome by utilizing reverse phase (RP)-MS and lipidomic analysis and to include other biological matricies such a urine and feces. These types of analysis could provide a more holistic understanding of the impact of nutritional interventions on metabolism and physiology. Lastly, while the findings of this discovery study can be explained and make sense biologically, it is recommended that discovery studies should be followed-up with a targeted approach to quantify findings and replicated in independent and larger cohorts.

## Conclusions

In the current study, untargeted metabolomics analyses were carried out to discriminate the polar plasma metabolome changes associated with a prescribed protein intake at RDA (0.8 g·kg^−1^·d^−1^) or 2RDA (1.6 g·kg^−1^·d^−1^) for 10 weeks. Post-intervention, two-way repeated measures ANOVA revealed metabolites of increased amino acid flux such as urea and creatinine were higher in the 2RDA diet compared to the RDA diet. Further metabolites were different between the two diet groups such as tryptophan and creatine and glutamine, which are components of the arginine and proline metabolism and nitrogen metabolism. These metabolites and pathways are indicative of changes in key aspects of skeletal muscle metabolism, which showed a good association with reduced maintenance of mass and function (7). Conversely, in the 2RDA group biomarkers of increased amino acid flux were increased, such as urea and uridine. Along with pathways these are involved in (glycine, serine, and threonine metabolism and aminoacyl-tRNA metabolism) allow us to speculate these alterations in metabolites are indicative of induction of tissue anabolism. Overall, this study demonstrated shifts in the metabolome induced by a higher protein diet is very limited in its metabolomic impact. Of those few metabolites that were statistically altered, it can be suggested that these are indicative of a possible induction of pathways supporting tissue anabolism and maintenance of muscle mass. In contrast, reducing protein intake to current minimum requirements resulted in a more catabolic metabolomic profile, potentially congruent with changes in skeletal muscle phenotype previously reported in the same cohort.

## Data Availability Statement

The authors can confirm we have deposited our metabolomics data to the EMBL-EBI MetaboLights database (doi: 10.1093/nar/gks1004. PubMed PMID: 23109552) with the identifier MTBLS1325.

## Ethics Statement

The study was reviewed and approved by the Southern Health and Disability Ethics Committee (New Zealand; 15/STH/236) and was conducted in accordance with the Declaration of Helsinki. This trial was registered on 3rd March 2016 at the Australia New Zealand Clinical Trial Registry (www.anzctr.org.au) at ACTRN 12616000310460.

## Author Contributions

CM, AM, SK, NR, AS, K-HW, and DC-S designed the research. CM, AM, SM, NZ, FR, and PS conducted the research. BD, CM, and KF conducted the statistical analysis. BD wrote the manuscript. DC-S had primary responsibility for the final content of manuscript. All authors provided content and feedback to the manuscript and read and approved the final manuscript.

### Conflict of Interest

SK, NR, and KF are current employees of AgResearch Ltd. The remaining authors declare that the research was conducted in the absence of any commercial or financial relationships that could be construed as a potential conflict of interest.

## Abbreviations

WHO: World Health Organization
RDA: recommended daily allowance
HILIC-MS: hydrophilic interaction liquid chromatography-mass spectrometry
BMI: body mass index
ANOVA: analysis of variance
BCAAs branched chain amino acids
TMAO: trimethylamine *N*-Oxide
CVD: cardiovascular disease.
