# Supplementary material for: Impact of a High Protein Intake on the Plasma Metabolome in Elderly Males: 10 Week Randomized Dietary Intervention
Source: Front Nutr. 2019 Dec 6;6:180. doi: 10.3389/fnut.2019.00180 (PMC6910071; doi:10.3389/fnut.2019.00180)
Supplement: Supplementary file 1 [file Data_Sheet_1.docx]

Supplementary table 1: Identification of significant features by 2-way repeated measures ANOVA

| Metabolite | m/z | Theoretical Mass | Ion | Rt (mins) | Ionisation mode | Class | Fragment | HMDB |
| --- | --- | --- | --- | --- | --- | --- | --- | --- |
| Urea | 61.0397 | 60.0553 | M+H | 7.41 | Positive | 1 |  | HMDB0000294 |
| Lenticin | 247.1439 | 246.1368 | M+H | 8.25 | Positive | 2 |  | HMDB0061115 |
| Indoleacrylic acid | 188.0694 | 187.0633 | M+H | 2.26 | Positive | 2 |  | HMDB0000734 |
| Glutarylcarnitine | 276.1435 | 275.1369 | M+H | 9.05 | Positive | 2 | 166.0720 | HMDB0013130 |
| Trigonelline | 138.0544 | 137.136 | M+H | 10.05 | Positive | 1 | 77.0791 | HMDB0000875 |
| TMAO | 151.1435 |  | 2M+H | 10.11 | Positive | 1 |  | HMDB0000606 |
| Tryptophan | 205.0969 | 205.0969 | M+H | 10.84 | Positive | 1 |  | HMDB0000929 |
| Isonipecotic acid | 130.0859 | 130.0862 | M+H | 11.01 | Positive | 1 |  |  |
| Proline | 116.0706 | 115.1305 | M+H | 11.04 | Positive | 1 |  | HMDB0000162 |
| Creatinine | 114.0660 | 114.0662 | M+H | 11.20 | Positive | 1 |  | HMDB0000562 |
| Methylimidazoleacetic acid | 141.0656 | 140.0586 | M+H | 11.39 | Positive | 2 |  | HMDB0002820 |
| Uric Acid | 169.0389 | 168.1103 | M+H | 11.48 | Positive | 2 | 119.01 | HMDB0000289 |
| Dihydrothymine | 129.0656 | 128.1292 | M+H | 12.82 | Positive | 2 | 88.0393 | HMDB0000079 |
| Creatine | 132.0763 | 132.0768 | M+H | 12.58 | Positive | 1 |  | HMDB0000064 |
| Threonine | 120.0683 | 120.0655 | M+H | 13.16 | Positive | 1 |  | HMDB0000167 |
| Glycoyamine | 118.0859 | 118.0867 | M+H | 13.29 | Positive | 1 |  | HMDB0000128 |
| Uridine | 244.0654 | 243.0617 | M-H | 8.47 | Negative | 1 |  | HMDB0000296 |
| 2-Aminoadipic acid | 160.0607 | 160.0609 | M-H | 13.19 | Negative | 1 |  | HMDB0000510 |
| Glutamine | 145.0610 | 145.0613 | M-H | 13.36 | Negative | 1 | 112.0401 | HMDB0000641 |
| Glutamic Acid | 146.0649 | 146.0453 | M-H | 13.66 | Negative | 1 |  | HMDB0000148 |
| Arginine | 173.0086 | 173.1039 | M-H | 13.79 | Negative | 1 |  | HMDB0000517 |
| Dimethylglycine | 102.0554 | 103.0663 | M-H | 13.82 | Negative | 2 |  | HMDB0000092 |
| Unknown M145T531 | 145.1049 |  |  | 8.85 | Positive | 4 |  |  |
| Unknown M160T639 | 160.0969 |  |  | 10.64 | Positive | 4 |  |  |
| Unknown M205T653 | 205.03 |  |  | 10.87 | Negative | 4 |  |  |

m/z, mass-to-charge ratio

Class 1: Identified marker confirmed by an authentic standard

Class 2: putatively identified marker based on elemental composition

Class 4: Unknown compound

Four levels of identification based on Metabolomics Standard Initiative


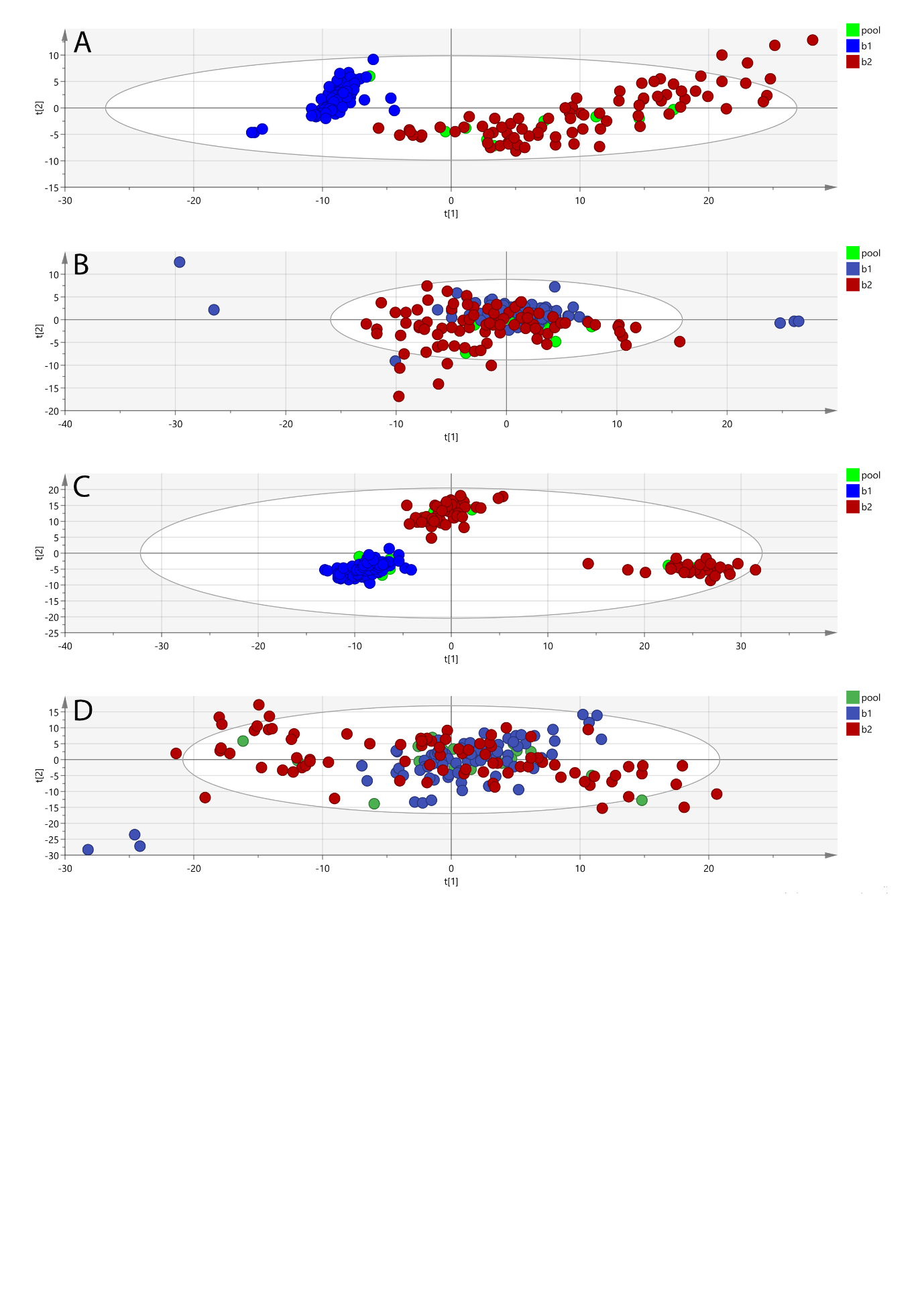


**New figure and caption**

**
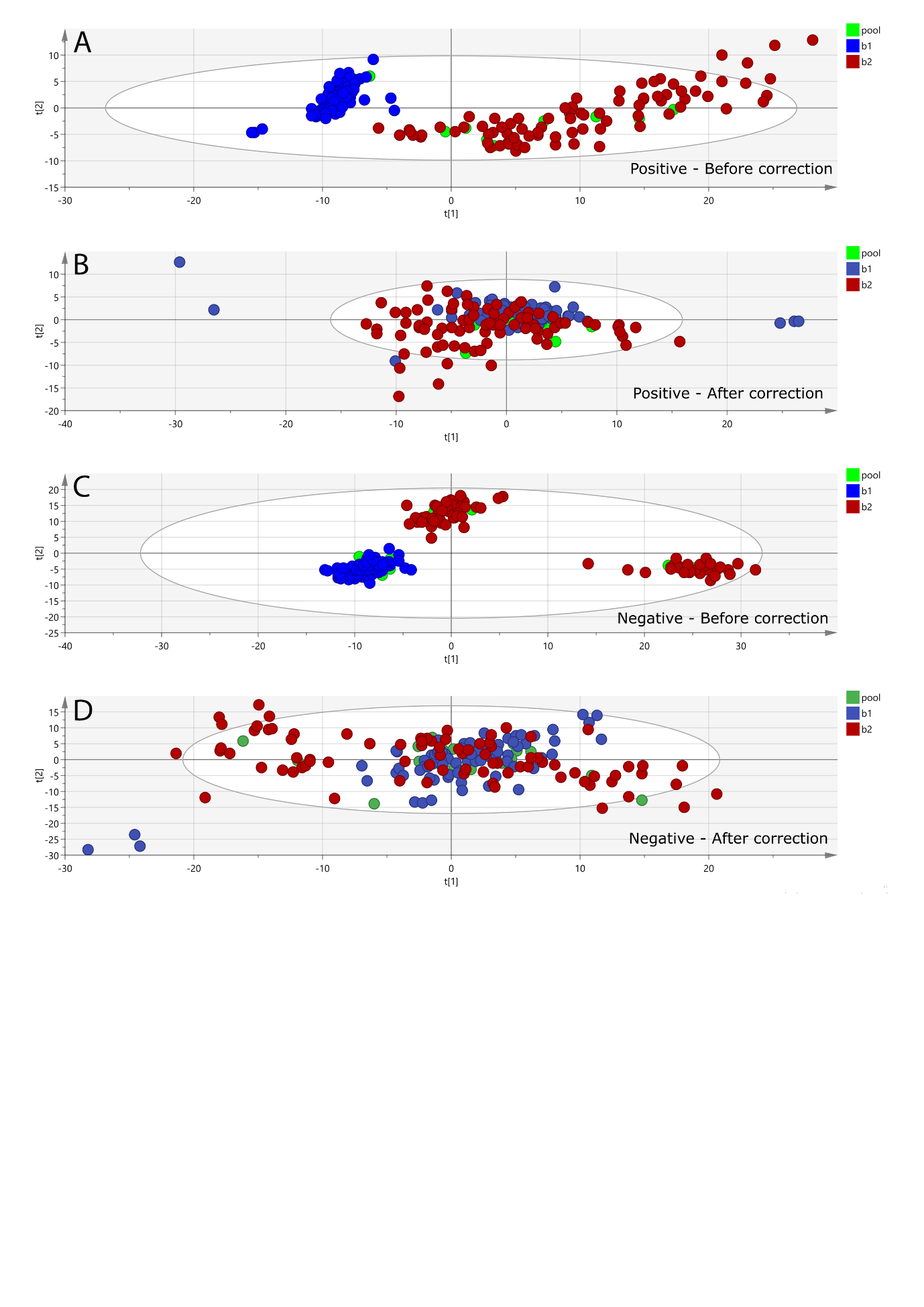
**

**Supplementary figure 1: Principal compenent analysis (PCA score plots).** PCA before batch correction in postive mode (A) and negative mode (C). A Lowess algorithim was applied utilising regularly measured pooled QC samples to correct for run-order and batch effects, resulting in the removal of batch differences and tight clustering of samples and pooled QC’s for both postive mode (B) and negative mode (D).


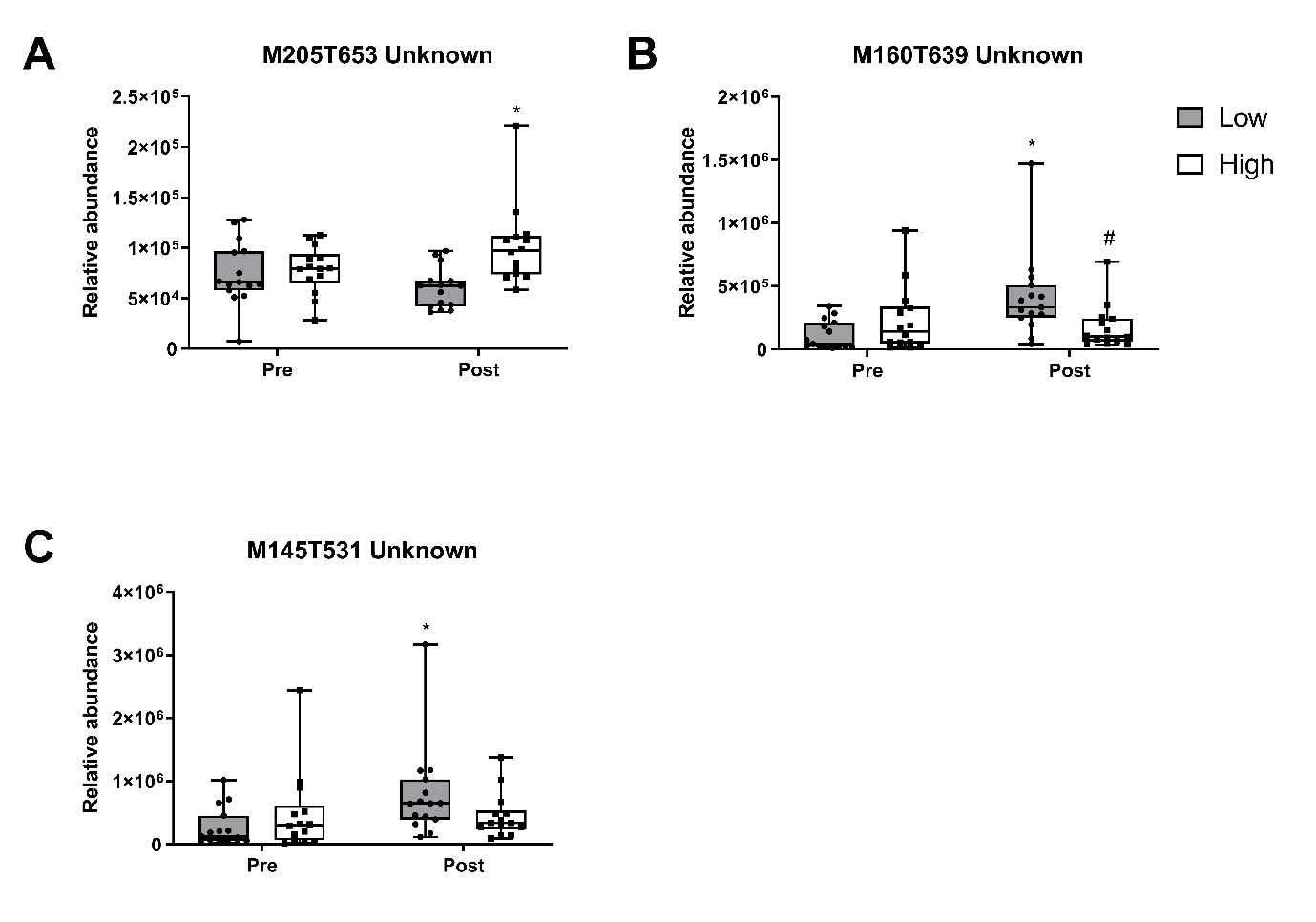


Supplementary figure 2: **Unidentified significant metabolites.** Box and whisker plot show mean, first, and third quartiles, and maximum and minimum values. # denotes significant differences of metabolites between 2RDA and RDA. * represents significant differences of metabolites between pre and post intervention within each group. (A) M205T653, m/z 205.0347, retention time 10.8787 mins and detected in negative ionisation mode. (B) M160T639; m/z 160.0969, retention time at 10.6432 mins and deteceted in positive ionisation mode. (C) M145T531; m/z of 145.1049, retention time of 8.8503 mins and detected in positive ionisation mode.
